# Editorial: New strategies for spinal cord injury and immunotherapy targeting novel programmed death pathways

**DOI:** 10.3389/fnins.2025.1689295

**Published:** 2025-09-24

**Authors:** SongOu Zhang

**Affiliations:** School of Medicine, Ningbo University, Ningbo City, Zhejiang Province, China

**Keywords:** spinal cord injury, immunotherapy, programmed death pathways, strategies, editorial

## Introduction

The complex pathophysiology of spinal cord injury (SCI) has long posed significant therapeutic challenges, with limited clinical options available for functional recovery. This Research Topic brings together cutting-edge research exploring novel programmed cell death pathways—particularly ferroptosis and cuproptosis—and their therapeutic targeting through immunotherapy, stem cell therapy, and natural compounds. The seven studies herein collectively advance our understanding of secondary injury mechanisms and unveil promising translational strategies.

## Reimagining programmed cell death in SCI pathogenesis

Traditionally, SCI research focused on apoptosis and necrosis as primary drivers of neuronal loss (Aita et al., 2024). This Research Topic expands the paradigm by highlighting iron-dependent ferroptosis and copper-mediated cuproptosis as critical amplifiers of secondary injury. Ferroptosis, characterized by lipid peroxidation and glutathione peroxidase 4 inactivation, exacerbates neural damage through reactive oxygen species accumulation and mitochondrial dysfunction (Guo et al., 2025). She et al. comprehensively reviews 15 natural compounds that inhibit ferroptosis by activating Nrf2/HO-1 signaling or modulating GPX4/SOD2 expression. Similarly, Xu et al. establishes cuproptosis—driven by copper overload and FDX1-mediated proteotoxic stress—as a key contributor to SCI pathology, with therapeutic potential in copper chelation and chaperone regulation.

It is important to note that these emerging forms of programmed cell death do not occur in isolation but exhibit intricate crosstalk with classical apoptotic and necrotic pathways (Atkin-Smith, 2021). For instance, mitochondrial dysfunction—a hallmark of apoptosis—can also trigger ferroptotic and cuproptotic cascades, while inflammatory necrosis (necroptosis) may synergistically amplify lipid peroxidation in ferroptosis (Guo et al., 2025; Xiong et al., 2025). Such interplay suggests a complex network of cell death modalities that collectively contribute to neuronal loss and glial damage in SCI. Therapeutic strategies targeting one form of cell death may inadvertently affect others, underscoring the need for a holistic understanding of their convergence in spinal cord pathology (Song et al., 2024).

## Immunomodulation: beyond checkpoint inhibition

While PD-1/PD-L1 checkpoint blockade revolutionized cancer immunotherapy, its role in neuroinflammation remains nuanced (Lin et al., 2024). Wu et al. reveals a dual neuroprotective/neurotoxic role for PD-L1/PD-1 signaling in SCI, where inhibition may reduce neuronal apoptosis but exacerbate inflammation. Bibliometric analysis underscores the need for stage-specific modulation, as PD-1 expression dynamically shifts during injury progression.

This duality presents substantial challenges for clinical translation. The narrow therapeutic window of PD-1 inhibition—potentially beneficial in early phases but harmful in later inflammatory stages—complicates timing and dosing in clinical trials. Moreover, efficacy appears limited when intervention is delayed, as chronic SCI may exhibit entrenched inflammatory networks less responsive to checkpoint modulation. Variability in patient immune status and injury heterogeneity further necessitate biomarker-guided patient stratification to identify those most likely to benefit from PD-1/PD-L1-targeted therapies.

Guan et al. further links METTL3-mediated m6A RNA modification to NLRP3 inflammasome activation, suggesting epitranscriptomic regulation as a novel immunotherapeutic target for pyroptosis control in SCI.

## Stem cells: multimodal regulators of cell death

Shen et al. highlights stem cells as “multimodal therapeutics” against ferroptosis. Mesenchymal stem cells and their exosomes restore redox balance by transferring functional mitochondria, delivering non-coding RNAs (e.g., miR-26a-5p), and upregulating GPX4/xCT. When combined with biomaterials, stem cells synergistically scavenge ROS and modulate synaptic stability. Arzhanov et al. reinforces this by demonstrating miR-20a inhibition in neural stem cells enhances STAT3/PI3K survival pathways under oxidative stress.

Despite these promising mechanisms, significant challenges impede the clinical translation of stem cell therapy. A major issue is cell heterogeneity—different sources yield MSCs with varying secretory profiles and therapeutic potentials (Liu et al., 2025). Poor survival and engraftment of transplanted cells in the hostile inflammatory microenvironment of SCI remain critical obstacles. Immune rejection, though reduced with allogeneic MSCs due to their immunomodulatory properties, still necessitates careful matching or immunosuppressive regimens. Timing of intervention is also crucial; while subacute phases may offer a more conducive environment for cell engraftment, chronic stages might require combinatorial approaches to modulate scar formation and inflammation.

Opportunities lie in bioengineering and combination strategies. Biomaterial scaffolds can enhance stem cell retention, survival, and directed differentiation at the lesion site. Genetically modifying stem cells to overexpress anti-ferroptotic factors or immunomodulatory cytokines could boost efficacy. Moreover, exosome-based therapies—derived from stem cells—offer a cell-free alternative with reduced risks of tumorigenicity and immunogenicity, alongside better storage and handling properties.

## Clinical translation: challenges and horizons

Despite promising preclinical data, clinical efficacy remains variable. Wu et al. notes inconsistent outcomes in PD-1 inhibitor trials for SCI, while Shen et al. cites phase I/III studies where MSC-exosomes improved ASIA scores in subacute—but not chronic—SCI. Key barriers include stem cell heterogeneity, timing of intervention, and immune rejection. Future work must prioritize: **1. Pathway crosstalk**: how ferroptosis, cuproptosis, and apoptosis intersect in SCI. **2. Combination therapies**: biomaterial-enhanced stem cell delivery with immunomodulators. **3. Patient stratification**: biomarker-guided therapy. Regarding patient stratification, specific biomarkers are emerging to tailor therapies. For immunomodulatory approaches such as PD-1/PD-L1 inhibition, CSF levels of soluble PD-1 or peripheral CD4+/CD8+ T cell ratios may help identify patients with active immune engagement who are more likely to respond. In stem cell therapy, MRI-based lesion characteristics and serum levels of inflammatory markers can predict engraftment and functional recovery. Genetic profiling, such as polymorphisms in Nrf2 or GPX4, might also indicate susceptibility to ferroptosis and response to antioxidant therapies (Luan et al., 2023). Integrating multimodal data—imaging, molecular biomarkers, and clinical scores—into algorithms could enable precision medicine for SCI, ensuring the right therapy is delivered to the right patient at the right time.

## Concluding remarks

This Research Topic illuminates programmed cell death not as an endpoint, but as a dynamic network amenable to therapeutic reprogramming. As we unravel the “dialogue” between immune cells, neurons, and glia in the injured spinal cord, the horizon of neuroimmunotherapy broadens with transformative potential.
